# A noninvasive mucinous cystic neoplasm with intermediate-grade dysplasia of the pancreas and extensive squamous metaplasia: a case report with clinicopathological correlation

**DOI:** 10.1186/1746-1596-7-89

**Published:** 2012-07-31

**Authors:** Peifeng Li, Yingmei Wang, Qingqing Zhang, Yixiong Liu, Yang Lv, Zhe Wang

**Affiliations:** 1 Department of Pathology, State Key Laboratory of Cancer Biology, Xijing Hospital and School of Basic Medicine, Fourth Military Medical University, Xi’an, Shaanxi, 710032, People's Republic of China; 2 Department of Pathology, The General Hospital of Jinan Military Command, Ji’nan, China; 3 Department of Hepatobiliary Surgery, Weifang People's Hospital, Weifang, China

**Keywords:** Mucinous cystic neoplasm, Squamous metaplasia, Pancreas, Immunohistochemistry

## Abstract

**Abstract:**

Squamous metaplasia presenting in noninvasive mucinous cystic neoplasm (MCN) of the pancreas is extremely rare. We described a case of 39-year-old Chinese female with a 5-year history of a slow growing mass in the left upper abdomen and an 18-month history of surgical incision exudation. The patient underwent cystojejunostomy, laparotomy and distal pancreatectomy consecutively because of the initial diagnosis of “pancreatic cyst”. The histological section showed columnar mucin-producing epithelium formed small papillary projections and extensively visible squamous metaplasia. Therefore the diagnosis of “noninvasive MCN with intermediate-grade dysplasia of the pancreas and extensive squamous metaplasia” was made finally. The squamous component of the pancreas may be derived from pluripotent stem cells, and may be in association with cystojejunostomy.

**Virtual slides:**

The virtual slide(s) for this article can be found here http://www.diagnosticpathology.diagnomx.eu/vs/1322364365718540

## Background

Mucinous cystic neoplasm (MCN) is a rare exocrine pancreatic tumor that can be classified into four histopathological types [1]: noninvasive MCN with low-grade, intermediate-grade or high-grade dysplasia and invasive MCN. MCN is composed of columnar mucin-producing epithelial layers supported by a distinctive ovarian-type stroma. Typically, MCN occurs almost exclusively in the body and/or the tail of the pancreas in perimenopausal women and shows no communication with the pancreatic ductal system. Extensive squamous metaplasia presenting in noninvasive MCN of the pancreas is extremely rare, although focal squamous metaplasia of the pancreatic ductal columnar cells can be observed in cases with inflammation [2]. In this report, we describe a unique case of noninvasive MCN with intermediate-grade pancreatic dysplasia and extensive squamous metaplasia. In addition, we discuss the pathogenesis of squamous metaplasia and its clinicopathological correlation.

## Case presentation

### Clinical history

**Figure 1:**
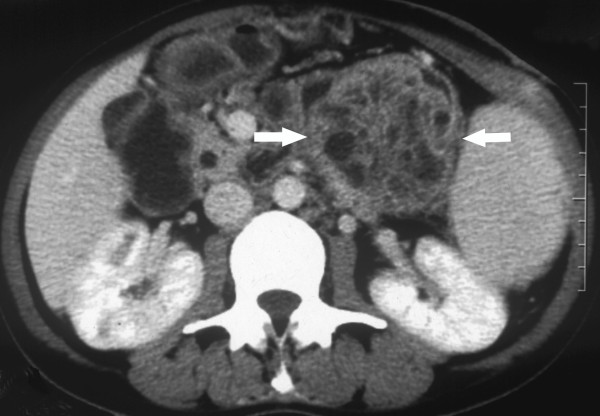
**Abdominal CT scan.** The tumor showing a heterogeneous multiloculated cystic mass with low density in the tail of the pancreas (white arrow), compressing the adjacent organs.

A 39-year-old Chinese female was referred to our hospital with a 5-year history of a slow growing mass in the left upper abdomen and an 18-month history of surgical incision exudation. The painless mass was found incidentally with an initial diagnosis of pancreatic cyst 5 years previously, and the palliative bypass procedure of cystojejunostomy was performed. However, due to the increasing size of the mass, the patient underwent a laparotomy 2 years later. This revealed a pancreatic tumor which was inoperable because of the major adhesion surrounding the neoplasm, stomach, greater omentum, mesentery and abdominal wall. Some of the anastomotic stoma tissue was excised for pathological examination, and a diagnosis of noninvasive MCN with intermediate-grade dysplasia was made. After 18 months, the surgical incision began to produce exudate. On admission to hospital, physical examination revealed a single, deep-seated, painless mass and two incisional sinuses with exudation in the left upper abdomen, without tenderness or muscular tension. Laboratory investigations were unremarkable, and serum levels of carbohydrate antigen 19–9 and carcinoembryonic antigen were within normal ranges. Abdominal ultrasonography and computer tomography scan revealed a 7.8 cm × 7.3 cm, heterogeneous hypoechoic or low-density mass with poorly defined margins in the tail region of the pancreas, compressing other adjacent organs. The mass was composed of several large loculi with an irregular thickening of the cyst wall and papillary excrescences projecting into the cystic cavity (Figure 1). Splenomegaly was also found. The patient underwent a distal pancreatectomy and splenectomy, during which a pseudo-encapsulated cystic mass adhering to the greater curvature of stomach and distal duodenum was observed. The pancreatic parenchyma in the region of the cyst was completely atrophied, and the previously performed anastomosis was obliterated. After an uneventful postoperative recovery, the patient remained symptom-free without recurrence during the 14-month follow up.

### Pathologic Findings

The resected neoplasm measuring 7.8 cm × 7.3 cm × 6.5 cm presented as a round mass with a fibrous pseudocapsule of variable thickness. In cross-section, the specimen revealed a multilocular tumor with cystic spaces ranging in size from a few millimeters to 1.3 centimeters in diameter, and containing grey–tan cloudy gelatinous material. The internal surface of the lumina showed multiple papillary projections and mural nodules. The spleen was intumescent and free of tumor invasion.

**Figure 2:**
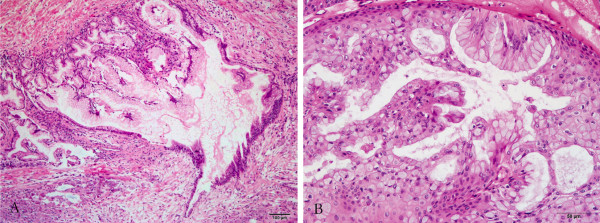
**Histopathology finding of tumor. (A)** The hyperplastic columnar epithelium characterized by basally located nuclei forming small papillary projections with crowding of slightly enlarged nuclei in the stoma tissue sample. Crypt-like invaginations can be found focally (H&E, 100×). **(B)** Foci of squamous metaplasia and the glandular element are present in the neoplasm. Squamous cells show slight or moderate nuclear hyperchromasia, enlargement and pleomorphism (H&E, 200×).

The specimen that was resected at the anastomotic stoma 3 years previously showed two distinct components: an inner epithelial layer and an outer cellular ovarian-type stromal layer. The columnar mucin-producing epithelium with pseudopyloric-type intracellular mucin and goblet cells formed small papillary projections – local pseudostratifications with crowding of slightly enlarged nuclei that are oriented perpendicular to the basement membranes. The columnar cells with bland uniform histological pattern and minor architectural atypia, were characterized by basally located nuclei and abundant supranuclear mucin which was positive for periodic acid Schiff with diastase and Alcian blue stains. Crypt-like invaginations were found focally (Figure 2A). The ovarian-type stroma, characteristic subepithelial tissue, was composed of densely packed spindle-shaped cells with elongated nuclei and sparse cytoplasm. Hypocellular and hyalinized connective tissue was present in variable amounts accompanied by focal lymphocytic infiltration. In addition to the typical features of MCN as discussed above, there were some unusual findings, including extensive pronounced squamous metaplasia and an obviously decreased stroma in the complete resection specimen (Figure 2B). Squamous epithelium combined with the top glandular epithelium excreting mucin. The stratified squamous epithelium formed papillary excrescences with sparse hyalinized fibrovascular stromal cores that protruded into cysts. The tumor-to-stromal interfaces were smooth with no invasion by the tumor cells. The squamous epithelium in the basal layer exhibit moderate pleomorphism and contain large hyperchromatic nuclei with a high nucleus to cytoplasm ratio, and mitotic figures are more abundant than usual, but they never reached the level of that seen in carcinoma. The abundant cytoplasm was mostly eosinophilic but occasionally clear. Cytoplasmic borders between tumor cells were distinct. Intercellular bridges were easily identified in the vast majority of tumor cells, and keratinization was not a prominent feature. Abundant ectatic vessels and focal neutrophils and eosinophils were observed in the rare stroma of the glandular element region. The tumor has a relatively well-demarcated pushing border.

**Figure 3:**
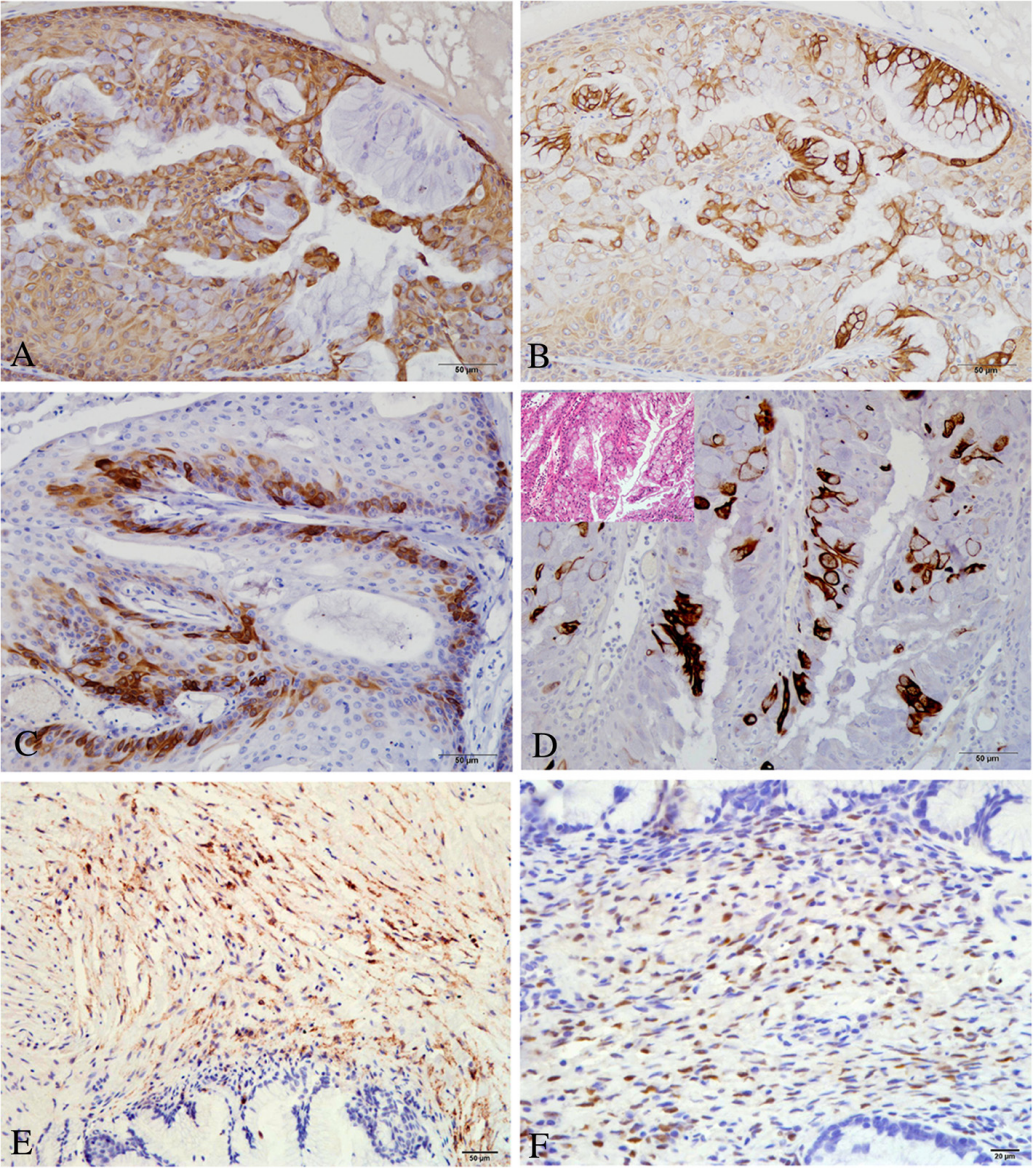
**Immunostaining of the tumor epithelium and stroma. (A)** The squamous cells were positive for CK5/6 (IHC, 200×). **(B)** The glandular epithelium was immunoreactive with LCK (IHC, 200×). **(C)** CK14 was expressed in some basal cells (IHC, 200×). **D)** Some cells in the micropapillary proliferation architectures were immunoexpressed with CK20 (IHC, 200×). The ovarian-type stroma was positive for CD10 **(E)** (IHC, 200×) and PR (F) (IHC, 400×).

In terms of immunohistochemistry (IHC), the epithelial component with squamous differentiation was strongly immunoreactive with high molecular weight cytokeratin (HCK, 34βE12) and CK5/6 (Figure 3A), while the glandular epithelial cells were strongly positive for low molecular weight CK (LCK, 35βH11) (Figure 3B). CK14 was expressed in some basal cells, but not in all of the squamous epithelium (Figure 3C). Some cells in the micropapillary proliferation architectures were immunoexpressed with CK20 (Figure 3D), while both squamous epithelium and glandular epithelium were negative for CK20. The expression of P63 was diffusely positive for the squamous cells with a tendency for intense positivity in the basilar part, but it was negative for the glandular epithelium. P53 positivity was found in the area of squamous epithelium (approximately 35% stained tumor cell nuclei), while glandular epithelium was negative (i.e. <10% stained tumor cell nuclei). The distinctive fusiform ovarian-type stroma showed positive staining for smooth muscle actin, CD10 (Figure 3E), estrogen receptor and progesterone receptor (PR) (Figure 3F). (All antibodies from Dako).

Electron micrography of the squamous differentiated region demonstrated some tonofibril intracytoplasm and a few intercellular desmosomes arranged on the lateral sides of plasma membranes.

## Discussion

MCN is a rare but distinctive pancreatic cystic neoplasm, defined by the lack of communication with the pancreatic duct system and the presence of a mucinous epithelium usually supported by an ovarian stroma. In our patient, the distinction from other reported typical MCNs was the extensive squamous metaplasia. This feature might be an indication of a longstanding MCN in which gradual replacement of the mucinous epithelium by squamous epithelial cells occurred. The pathogenesis is unclear, and there are two theories [3]: (1) the squamous element is derived from squamous transdifferentiation of the adenomatous element; (2) the squamous element of the pancreatic tumor arises from pluripotent stem cells. In our case, the adenoma and squamous elements were mixed, and a gradual transition was identified. The glandular epithelial cells with LCK immunoreaction tended to be located on the surface of the squamous element in the area of significant hyperplasia. We speculate that the squamous component of the pancreas may be derived from pluripotent stem cells, and may be induced in our case by cystojejunostomy and drainage. Furthermore, an inflammatory reaction was found both in the anastomotic stoma biopsy and complete resection specimens, and the squamous metaplasia may be related to the inflammation.

The preoperative diagnosis of MCN is very important, since other types of pancreatic neoplasm may be treated differently and the grade of neoplasm does accurately predict the outcome [4,5]. The main differential diagnosis includes other neoplastic cystic lesions (serous cystic neoplasm and the intraductal papillary mucinous neoplasm) and non-neoplastic cystic lesions. MCN must be distinguished in particular from an inflammatory pseudocyst, because drainage is appropriate for the latter, but long-standing drainage can transform apparently histologically benign MCNs into invasive MCNs [6,7]. In our patient, the tumor also needed to be differentiated from a squamoid cyst of pancreatic ducts and mixed-epithelial papillary cystadenomas of borderline malignancy of mullerian type with squamous overgrowth (MEBMMSO). A squamoid cyst of pancreatic ducts usually has a thin wall lined by transitional/squamous cells without keratinization or a granular layer. The cyst is more commonly unilocular and typically contains distinctive acidophilic concentric enzymatic concretions which indicate their communication with the acinar system [8]. MEBMMSO was defined as an ovarian cystic tumor composed of major squamous intracystic fronds accompanied by minor epithelial components of mullerian types, such as mucinous, serous, and endometrioid, and prominent intraepithelial infiltration by neutrophilic leukocytes [9]. However, to date, this has not been reported in the pancreas, and there was no squamous element in the stoma biopsy in our patient. Lymphoepithelial cyst (LEC) of the pancreas may also present as pancreatic multilocular mass with cysts lined by squamous epithelium in the tail. However, it was characterized by cysts, some containing keratin, and lined by mature stratified squamous epithelium surrounded by dense lymphoid tissue, often with prominent follicles [10]. LEC lacks the ovarian-type stroma, furthermore, papillary changes and mucinous cells are exceedingly rare, and if present, are very focal. Although it has been demonstrated that endoscopic ultrasonography can provide detailed images of internal structures and can be effective for the diagnosis of MCN [11], the best approach to obtain an accurate preoperative diagnosis is the comprehensive evaluation of all available clinical, serological, radiological, and pathological findings [12].

All MCNs should be resected to prevent malignant transformation but can be monitored for an appropriate time if the lesion is small without the presence of mural nodules [13-15]. Our patient underwent a cystojejunostomy at the age of thirty-four years, presumably with an underlying misdiagnosis of a pancreatic pseudocyst without characteristic clinical finding except for an incidental mass. Two years later, the diagnosis of “noninvasive MCN with intermediate-grade dysplasia” was diagnosed using stoma tissue sample. The complete resected specimen showed extensive squamous metaplasia, distinct papillary proliferation, and obviously decreased stroma, which might indicate a poorer outcome [6]. Furthermore, IHC results showed that P53 nuclei expression increased in the squamous element, which may also suggest a poor prognosis [16]. More studies of series of patients with MCN and longer follow-up times are needed to explore whether extensive squamous metaplasia represents neoplasm progression and/or predicts a poor prognosis.

## Conclusions

We have described a case of 39-year-old Chinese female with a 5-year history of a slow growing mass in the left upper abdomen and an 18-month history of surgical incision exudation. The patient underwent cystojejunostomy, laparotomy and distal pancreatectomy consecutively because of the initial diagnosis of “pancreatic cyst”. The histological section showed columnar mucin-producing epithelium formed small papillary projections and extensively visible squamous metaplasia. Therefore the diagnosis of “Noninvasive MCN with intermediate-grade dysplasia of the pancreas and extensive squamous metaplasia” was made finally. The squamous component of the pancreas may be derived from pluripotent stem cells, and may be in association with cystojejunostomy and the inflammation. More studies of series of patients with MCN and longer follow-up times are needed to explore whether extensive squamous metaplasia represents neoplasm progression and/or predicts a poor prognosis.

## Abbreviations

HCK: High molecular weight cytokeratin; IHC: Immunohistochemistry; LCK: Low molecular weight cytokeratin; MCN: Mucinous cystic neoplasm; MEBMMSO: Mixed-epithelial papillary cystadenomas of borderline malignancy of mullerian type with squamous overgrowth; PR: Progesterone receptor.

## Competing interests

The authors declare that they have no competing interests.

The research for this article was supported by grants from National Natural Science Foundation Of China (Nos. 30771120 and 81072103).

## Authors' contributions

PFL, YMW and ZW drafted the manuscript. QQZ was responsible for collecting the patient material. YXL and YL carried out the tissue staining, immunoassays, and electron microscopic examination. PFL and ZW conducted the analysis of the histological features and clinicopathological relations. All authors read and approved the final manuscript.

## Consent

Written informed consent was obtained from the patient for publication of this Case Report and any accompanying images. A copy of the written consent is available for review by the Editor-in-Chief of this journal.

## References

[B1] Fred TB FC, Ralph HH, Neil DT WHO Classification of Tumours of the Digestive System 2010 Lyon: IARC

[B2] Kimura W Pancreatic lithiasis and intraductal papillary-mucinous neoplasm with special reference to the pathogenesis of lithiasis J Hepatobiliary Pancreat Sci 2010 17 6 776 781 10.1007/s00534-009-0180-9 19779665

[B3] Anagnostopoulos GK Aithal GP Ragunath K Kaye P Rowlands BJ Squamous cell carcinoma of the pancreas: report of a case and review of the literature JOP 2006 7 1 47 50 16407618

[B4] Wilentz RE Albores-Saavedra J Zahurak M Talamini MA Yeo CJ Cameron JL Hruban RH Pathologic examination accurately predicts prognosis in mucinous cystic neoplasms of the pancreas Am J Surg Pathol 1999 23 11 1320 1327 10.1097/00000478-199911000-00002 10555000

[B5] Sarr MG Carpenter HA Prabhakar LP Orchard TF Hughes S van Heerden JA DiMagno EP Clinical and pathologic correlation of 84 mucinous cystic neoplasms of the pancreas: can one reliably differentiate benign from malignant (or premalignant) neoplasms? Ann Surg 2000 231 2 205 212 10.1097/00000658-200002000-00009 10674612 PMC1420988

[B6] Zamboni G Scarpa A Bogina G Iacono C Bassi C Talamini G Sessa F Capella C Solcia E Rickaert F Mariuzzi GM Kloppel G Mucinous cystic tumors of the pancreas: clinicopathological features, prognosis, and relationship to other mucinous cystic tumors Am J Surg Pathol 1999 23 4 410 422 10.1097/00000478-199904000-00005 10199470

[B7] Shimizu Y Yasui K Yamao K Ohhashi K Kato T Yamamura Y Hirai T Kodera Y Kanemitsu Y Ito S Yanagisawa A Possible oncogenesis of mucinous cystic tumors of the pancreas lacking ovarian-like stroma Pancreatology 2002 2 4 413 420 10.1159/000065090 12138231

[B8] Volkan Adsay N Cystic lesions of the pancreas Mod Pathol 2007 20 Suppl 1 S71 93 17486054 10.1038/modpathol.3800706

[B9] Nagai Y Kishimoto T Nikaido T Nishihara K Matsumoto T Suzuki C Ogishima T Kuwahara Y Hurukata Y Mizunuma M Nakata Y Ishikura H Squamous predominance in mixed-epithelial papillary cystadenomas of borderline malignancy of mullerian type arising in endometriotic cysts: a study of four cases Am J Surg Pathol 2003 27 2 242 247 10.1097/00000478-200302000-00014 12548172

[B10] Adsay NV Hasteh F Cheng JD Bejarano PA Lauwers GY Batts KP Kloppel G Klimstra DS Lymphoepithelial cysts of the pancreas: a report of 12 cases and a review of the literature Mod Pathol 2002 15 5 492 501 10.1038/modpathol.3880553 12011254

[B11] Yamao K Nakamura T Suzuki T Sawaki A Hara K Kato T Okubo K Matsumoto K Shimizu Y Endoscopic diagnosis and staging of mucinous cystic neoplasms and intraductal papillary-mucinous tumors J Hepatobiliary Pancreat Surg 2003 10 2 142 146 10.1007/s00534-002-0802-y 14505147

[B12] Visser BC Muthusamy VR Yeh BM Coakley FV Way LW Diagnostic evaluation of cystic pancreatic lesions HPB (Oxford) 2008 10 1 63 69 10.1080/13651820701883155 18695762 PMC2504857

[B13] Yamao K Yanagisawa A Takahashi K Kimura W Doi R Fukushima N Ohike N Shimizu M Hatori T Nobukawa B Hifumi M Kobayashi Y Tobita K Tanno S Sugiyama M Miyasaka Y Nakagohri T Yamaguchi T Hanada K Abe H Tada M Fujita N Tanaka M Clinicopathological features and prognosis of mucinous cystic neoplasm with ovarian-type stroma: a multi-institutional study of the Japan pancreas society Pancreas 2011 40 1 67 71 10.1097/MPA.0b013e3181f749d3 20924309

[B14] Mizutani S Nakamura Y Ogata M Watanabe M Tokunaga A Tajiri T A case of giant mucinous cystic neoplasm of the pancreas resected with laparoscopic surgery J Nihon Med Sch 2009 76 4 212 216 10.1272/jnms.76.212 19755797

[B15] Campbell F Azadeh B Cystic neoplasms of the exocrine pancreas Histopathology 2008 52 5 539 551 10.1111/j.1365-2559.2007.02856.x 17903202

[B16] Scarlett CJ Salisbury EL Biankin AV Kench J Precursor lesions in pancreatic cancer: morphological and molecular pathology Pathology 2011 43 3 183 200 10.1097/PAT.0b013e3283445e3a 21436628

